# Climatic and Environmental Factors Influencing COVID-19 Transmission—An African Perspective

**DOI:** 10.3390/tropicalmed7120433

**Published:** 2022-12-12

**Authors:** Allan Mayaba Mwiinde, Enock Siankwilimba, Masauso Sakala, Faustin Banda, Charles Michelo

**Affiliations:** 1 Graduate School of Public Health, Department of Epidemiology Ridgeway Campus, University of Zambia, Lusaka P.O. Box 50516, Zambia; 2 Department of Public Health, Mazabuka Municipal Council, Mazabuka P.O. Box 670022, Zambia; 3 Graduate School of Business, University of Zambia, Lusaka P.O. Box 50516, Zambia; 4 School of Engineering, Department of Geomatic Engineering, University of Zambia, Lusaka P.O. Box 50516, Zambia; 5 The National Remote Sensing Centre, Plot Number 15302 Airport Road, Lusaka P.O. Box 310303, Zambia; 6 Harvest Research Institute, Lusaka P.O. Box 51176, Zambia

**Keywords:** Africa, COVID-19, climatic factors, environmental factors, transmission

## Abstract

Since the outbreak of COVID-19 was decreed by the World Health Organization as a public health emergency of worldwide concern, the epidemic has drawn attention from all around the world. The disease has since spread globally in developed and developing countries. The African continent has not been spared from the pandemic; however, the low number of cases in Africa compared to developed countries has brought about more questions than answers. Africa is known to have a poor healthcare system that cannot sustain the emerging infectious disease pandemic. This study explored climatic and environmental elements influencing COVID-19 transmission in Africa. This study involved manuscripts and data that evaluated and investigated the climatic and environmental elements of COVID-19 in African countries. Only articles written in English were considered in the systematic review. Seventeen articles and one database were selected for manuscript write-ups after the review process. The findings indicated that there is evidence that suggests the influence of climatic and environmental elements on the spread of COVID-19 in the continent of Africa; however, the evidence needs more investigation in all six regions of Africa and at the country level to understand the role of weather patterns and environmental aspects in the transmission of COVID-19.

## 1. Introduction

By December 2019, there were a number of pneumonia cases in Wuhan, Hubei Province, China [1]. These illnesses had spread to 19 countries by 31 January 2020 when there were 11,791 confirmed cases, including 213 fatalities [2]. Three months after the initial report, the World Health Organization (WHO) proclaimed a Public Health Emergency of International Concern because it had spread globally [3]. The WHO first referred to the new virus as 2019-nCoV [4] while the International Committee for the Taxonomy of Viruses referred to it as SARS-CoV-2. The WHO declared that the 2019 novel coronavirus was officially known as coronavirus illness (COVID-19) on 11 February 2020 [4,5]. Over 630,601,291 people have been affected globally by the infectious disease COVID-19 as of 10 November 2022 [6].

Since the first confirmed case of COVID-19 was reported in Egypt on 14 February 2020, Africa, home to more than 1 billion people, has been tracking COVID-19 cases [7,8]. From sub-Saharan Africa, the first case was reported in Nigeria on 27 February 2020 in an Italian patient who had travelled to Nigeria by plane from Italy on 25 February 2020. Since then, the continent recorded an increase in the number of cases. As of 23 March 2020, there were 4,116,102 confirmed cases of COVID-19 from 55 African countries [9]. A total of 110,163 deaths were reported in Africa, although 5,599,955 immunizations were given out and 3,690,639 persons made a full recovery [10].

Although the number of COVID-19 cases and fatalities might appear to be lower in Africa than in Europe, the USA, and the Middle East, the impact of the epidemic has caused constraints in the health sector, causing limited diagnostic and manpower due to scarce resources with a higher need for social and economic recovery than in developed countries [11]. The possible strive towards the implementation of public health interventions does not always lower infection rates, but to some extent, it causes a huge social and economic impact that ultimately causes a violation of possible lockdowns in most African countries so individuals can access basic social needs [12].

To stop the spread of COVID-19, many global and local public health measures have been put in place, such as vaccinations, social distancing, masks, and lockdowns that allow for some ventilation in indoor spaces. However, in some places, these actions were seen as punishments because people were reluctant to get vaccinated during vaccination campaigns in Africa, to some extent the majority did not wear masks in public and did not follow lockdowns [10]. The massive movement of people from and between regions and other parts of the world was reported to have increased the geographical distribution of COVID-19 [7].

Environmental and climatic factors like evaporation, humidity, gravity, wind speed, wind direction, and particulate matter have been shown to have a big effect on how respiratory droplets and aerosols spread [13].

COVID-19 has the potential to cause various lung complications, such as pneumonia and, in the most severe cases, acute respiratory distress syndrome, or ARDS, sepsis [14]. People who are exposed to air pollution for a long time are more likely to get sick and die from chronic diseases, which could make them more likely to get COVID-19 [15]. Some studies have established that climatic factors such as temperature, humidity, wind speed, and rainfall are some of the transmission influences of the novel COVID-19 disease whereas other infectious diseases, such as Middle East respiratory syndrome coronavirus (SARS), have differences in seasonal infection rates and deaths [16,17]. Most of the highest peaks of respiratory virus infections have been seen in the winter months [18]. High temperatures and high relative humidity can stop SARS-CoV from infecting people, but low temperatures and low humidity can keep the virus alive on contaminated surfaces for about two weeks [19]. Since COVID-19 spreads through close personal contact between individuals [20], it is important to comprehend the common climatic and environmental factors [21] that influence COVID-19 transmission throughout African nations [22]. Further studies are being carried out to establish the relationship between the climate and environmental factors in association with COVID-19 virus mutation [13]. The parallels, trends, and patterns of the pandemic in the 55 members of the African Union (AU) have not yet been thoroughly examined [23]. There is a lack of understanding of the environmental, climatic, social, and cultural factors that can lessen the transmission of COVID-19 in the majority of modeling estimates, response suggestions, and pandemic transmission in Africa [23].

To improve the effectiveness of the current public health measures, this review intends to examine the literature on climatic and environmental factors that affect the transmission of COVID-19 in African nations.

## 2. Materials and Methods

### 2.1. Eligibility Criteria

Manuscripts published research papers and data that evaluated and investigated the environmental factors that influence the transmission of COVID-19 in Africa were included in the study. Due to the difference in the use of the official language, only articles written in English were considered.

### 2.2. Literature Identification and Data Extraction

Two different authors were responsible for independently identifying relevant literature and extracting relevant data. Where there was disagreement, a third party was consulted to determine the article’s eligibility. A literature search was undertaken using the terms ‘COVID-19’, ‘coronavirus’, and ‘COVID-19’ as well as other conjugations of the terms ‘environmental variables’ and ‘Africa’ [24]. The titles of each manuscript were used to identify and extract information. After determining the title and determining that the content appeared to discuss environmental indicators influencing the spread of COVID-19 in any of the African countries or at the global level where Africa is included, information was obtained, and its full reference included the author, year, title, and abstract for further evaluation.

## 3. Results

### 3.1. Electronic/Manual Search

The systematic review of the Elsevier and Google Scholar Advanced Search databases generated a total of 17,268 articles. A total of 16,138 records were removed by automation tools, and 55 records were removed for other reasons. Elsevier returned 202 results whereas Google Scholar returned 875. Further evaluation was done where a total of 1077 records were evaluated, and 950 were eliminated because they did not meet the eligibility requirements; they did not study environmental issues. A total of 127 articles were requested for retrieval, of which, 61 were not recovered because they lacked environmental elements.

**Figure A1:**
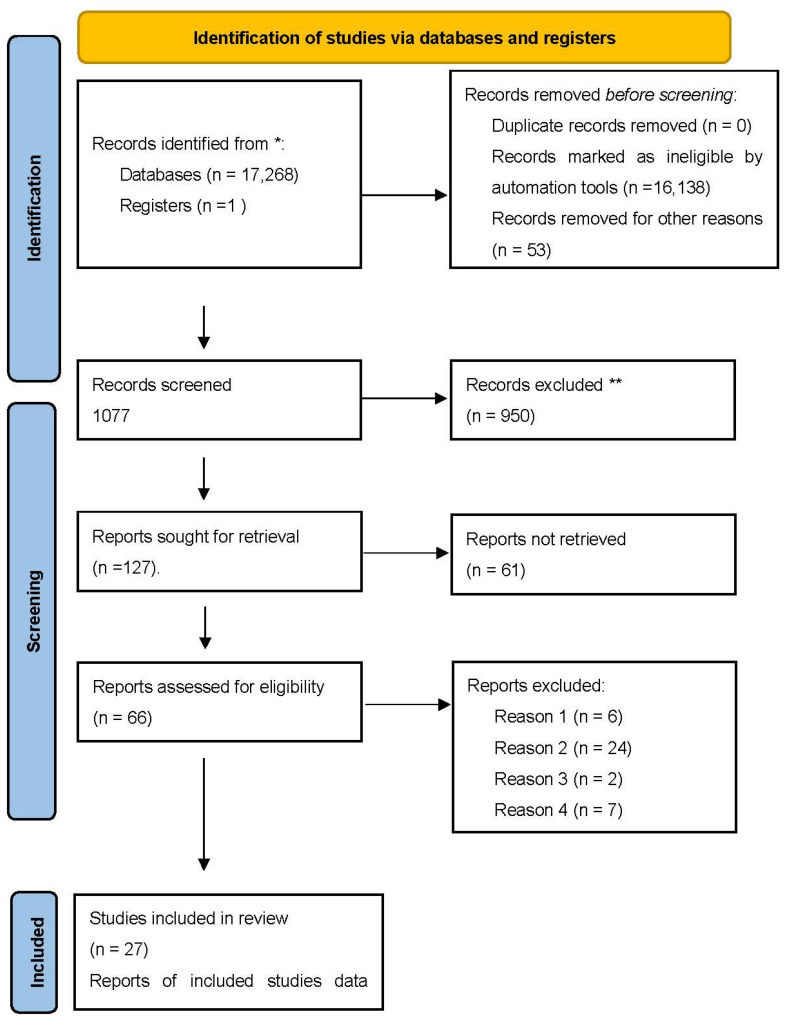
PRISMA Flow Diagram of Literature identification and selection. Literature identified * (database 17268, register 1), Records excluded ** 950. The final records from data bases 27 and 1 register.

The articles that passed the title and abstract screening (n = 66) were subjected to a full screening by investigators, which resulted in the exclusion of a total of 39 reports: (n = 6) reviews, (n = 24) does not analyze environmental reasons and Africa, (n = 2) letters to the editor, and (n = 7) duplicates. After the full-text screening, twenty-seven articles (n = 27 articles and 1 database) were included (n = 27 articles and 1 database) (Appendix A). Figure A1 shows a summary of the search procedure. 

### 3.2. COVID-19 Epidemic in Africa from 2020 to 2022

**Figure 1:**
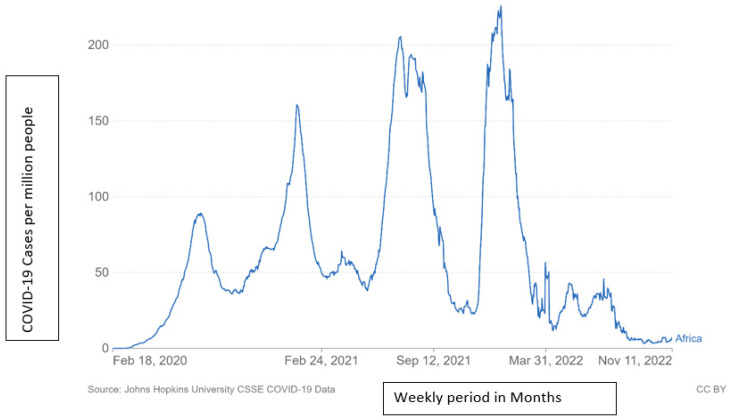
This figure is the daily COVID-19 documented cases per million persons in Africa between February 2020 and 11 November 2022.

**Figure 2:**
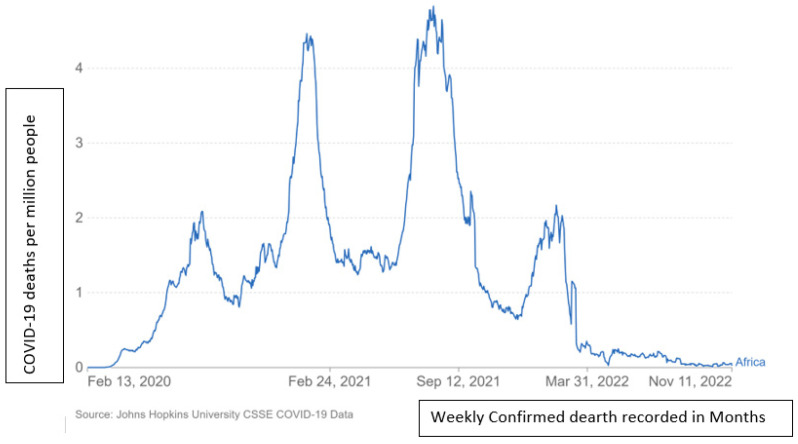
This figure indicates the daily confirmed deaths due to COVID-19 per million people in Africa from February 2020 to November 2022.

From the electronic database of John Hopkins University, Figure 1 below indicates the prevalence of COVID-19 in Africa from February 2020 to November 2022. Figure 2 indicates the mortality recorded in the same period from February 2020 to 11 November 2022 [25].

During the initial wave of the pandemic in Africa, the transmission of the COVID-19 virus was rather slow between February and May 2020. (Figure 1). The ensuing spike was first observed at the end of March and beginning of April when 2.6 million cases were rerecorded with the largest peak occurring on 24 July 2020 when 92.57 million cases were reported in African communities (Figure 1). There was an increase in November with a peak of 68.40 million instances recorded on November 30. A large increase was noticed on 1 December 2020 with the highest peak of the second wave of the epidemic curve reaching 166.89 million cases on 11 January 2021. The second curve reached its lowest point on 5 March 2021 with 47.74 million cases. In April 2021 the number of cases increased to 66.59 before declining to 39.48 on 16 May 2021, signaling the beginning of the third wave, which peaked on 15 July 2021 with 213.56 instances. Towards the end of the month, however, there was an increase to 171.56 million cases and 201.49 cases on 3 August, prior to a sharp decline. On 27 September 2021, there were 57.55 reported cases. November’s lowest number in the third wave was 3.41 million, and November’s greatest number was 4.70 million before recording 3.46 million, marking the beginning of the fourth wave, which reached 31.4 million by 31 December 2021. The infection rate on January 10, 2022, was the highest among the first, second, third, and fourth waves at approximately 33.51 [24]: the highest record since the COVID-19 pandemic. By the end of January, the number of COVID-19 cases had reached 20.73; by the end of February, it had reached 6.09 million [25].

### 3.3. COVID-19 Mortalities in Africa from 2020 to 2022

During the first wave of COVID-19, the death rate between 19 February and 20 March was below 0.01 (Figure 2). There was a spike on 28 July of 2.08 million deaths, and the highest spike was on 9 August 2021. The lowest before the second wave was 0.86, recorded on 5 October 2021. The highest spike in the second wave was 4.64 on 9 January 2021; there was a second spike of 4.60 on 24 January 2021. There was a slight increase from April to 7 June 2021 with a value of 1.42. The highest spike in the third wave of the first spike on 11 June 2021 was 4.55, and the second spike on 26 July 2021 was 4.82 deaths. The third spike during the third wave was on 29 July 2021 at 4.96, and the fourth recorded spike was on 4 August 2021 with 5.02 deaths. The first spike on 17 August 2021 was at 4.83 before dropping to 1.99 on 26 September 2021. October recorded 2.45 as the highest in 2021, which continued to decrease in November and had the highest of 0.93 and the lowest of 0.76 on 22 November 2021. On 13 December 2021, the lowest number of deaths recorded in the month was 0.67, which began to increase to 1.12 by 31 December. On 24 January 2022, the recorded number of deaths was 2.01, and February recorded 2.24 as the highest in the fourth wave before decreasing to 1.07 by 28 February 2022 [25].

### 3.4. Temperature, Humidity, and Wind Speed

Based on the analysis of the publications included in this review (Appendix B), it has been concluded that temperature is one of the major elements and the strongest environmental predictor of whether a region will have a smaller prevalence of COVID-19 cases [26,27]. A study was done in 16 Africa Union member states, including South Africa, Ghana, Nigeria, Egypt, Algeria, Morocco, Kenya, Congo, Côte d’Ivoire, Cameroon, Niger, Somalia, Tanzania, Zambia, Mozambique, and Madagascar [28]. The results showed that the temperature and the number of COVID-19 cases (r = −0.25, *p* = 0.001) and deaths (r = −0.18, *p* = 0.001) were linked in a statistically significant way. Moreover, a regression analysis showed that the number of cases and deaths dropped by 15.1% and 10.5%, respectively, for every 1 °C increase in temperature [28].

According to studies, regions in Zambia with temperatures above 22 degrees Celsius had a very low number of COVID-19 cases whereas those with temperatures below 22 degrees Celsius had a high number of cases [29]. When the daily mean temperature was below the global mean temperature, which is approximately 21.07 °C, the chance of COVID-19 transmission increased, according to global studies that included Africa [30]. In Mozambique, too, the number of confirmed cases of COVID-19 seems to be related to the temperature and pressure of the air [31].

Diouf et al. [32] found statistically significant inverse correlations between COVID-19 cases and temperature in the Maghreb and Gulf of Guinea. Niger and Mali, however, exhibited positive relationships [32]. Correlations with humidity and water vapor characteristics exhibited significant and positive values over the Sahelian and Gulf of Guinea countries and negative values over the Maghreb countries. The results also demonstrated that COVID-19 pandemic transmission is affected differently by the three climatic regions: (i) cold and dry environmental conditions; (ii) warm and humid environmental conditions; and (iii) cold and humid environmental conditions [32].

Other investigations indicated that the average temperature was statistically correlated negatively with the increase in confirmed cases of COVID-19 in Africa, but the average wind speed was statistically correlated favorably with the growth of COVID-19 [33].

According to [34], a 1 °C increase in average temperature was related to a 25.44% (95 CI: 1.12–3.65) decrease in COVID-19 growth in Africa. Similarly, a one percent increase in average wind speed was associated with a 22.13% (95 CI: 0.22–1.43) rise in confirmed COVID-19 cases in Africa [35].

When many cases were discovered in South Africa’s Western Cape (from 12 March 2020 to 30 June 2020), the average daily temperature was 15 °C, the relative humidity was 70%, the UV index was 7, and the wind speed was 17 km/h [36,37].

There was an average daily count of 343 confirmed cases in 127 African nations when the mean temperature was 20.5 °C, the relative humidity was 66.54%, and the wind speed was 1.94 m per second [38]. In some studies, issues related to temperature were still significantly negatively correlated with daily new cases when the temperature was below 20 °C [21] and positively correlated with daily new cases when the temperature was above 20 °C [29].

A global study revealed that the coefficients divulge a strong (positive and negative) association between weather conditions and COVID-19 infections in approximately 67.2% of the selected states [36]. In two African nations, Egypt and Ethiopia, a strong positive association was established between the number of new cases and temperature [35]. 

The number of daily new cases of COVID-19 was negatively correlated with temperature (r = 0.25), wind speed (r = 0.06), absolute humidity (r = 0.19), and relative humidity (r = 0.10) at the global level whereas the diurnal temperature range was positively correlated (r = 0.1) [38].

Other studies indicated that population size, average temperature, and median age do not have a significant impact on the number of cases when evaluated individually; nevertheless, their interactions with other determinants had a substantial effect on the transmission of COVID-19 [39].

It has been determined that countries located 1000 km closer to the equator can anticipate 33% fewer instances of COVID-19 per million population, all else being equal (given that a degree of latitude translates on average to 111 km). Changes occur in the number of COVID-19 cases per million for every degree of latitude shift [40].

Since temperature was substantially correlated with other climatic variables, such as solar irradiance and precipitation, this indicated that humidity would also have similar impacts even though it was not included as one of the explanatory factors in the datasets [29].

Note that we are not arguing that temperature is the only or main factor: in fact, there must be other conditions such as humidity, wind speed, air pressure and pollution which could also possibly play a role in the transmission of COVID-19, as temperature explains just 18% of the variability in disease spread [40]. 

It must be noted that the degree of influence differs in different situations and environments [41]. In Cape Town, South Africa, it was found that the influence of meteorological variables such as temperature and humidity is not always homogeneous, providing room for other intervening variables to have an influence [42]. Similarly, in Ghanaian studies, results show that wind speed and pressure have a positive linear relationship with the risk of COVID-19 spread whereas temperature and humidity have a nonlinear relationship [43].

The 16 African Union member states revealed a significant inverse correlation between humidity and the number of cases (r = −0.192, *p* 0.001) and deaths (r = −0.213, *p* 0.001). Furthermore, the regression results showed that with a 1% increase in humidity, the number of cases and deaths significantly reduced by 3.6% and 3.7%, respectively [28].

The influence of COVID-19 on relative humidity established that the cases of COVID-19 are oscillating between negative and positive from one country to another in four countries, namely South Africa, Morocco, Tunisia, and Ethiopia. The rise in relative humidity was associated with high COVID-19 infections in Morocco and Tunisia; however, the rise in relative humidity was associated with low cases in South Africa and Ethiopia. The relative humidity rises by 1 (%) in Morocco and 0.111 in Tunisia. The opposite was recorded in South Africa and Ethiopia when the relative humidity increased by 1% [44].

African countries have recorded that a 1% increase in average wind speed (m/s) is associated with an 11.21% (95 CI: 0.51–1.19) increase in confirmed cases of COVID-19 in Africa [34].

Wind speed has been recognized to play a critical role in the dispersal of bacterial and viral pathogens through the troposphere. The long-range transmission of viruses and bacteria contributes to increasing their distribution ranges in dormant or inactive states; this happens when more viruses are attached to the smallest airborne organic particles at heights of 3 km (Reche et al. [45]). Using the spatiotemporal credence for the exposure-response curve of meteorological factors and COVID-19 growth in Africa using the generalized additive model (GAM), the findings suggested that mean temperature and average wind speed are inversely related to the growth curve of COVID-19 in Africa [34].

### 3.5. COVID-19 and UV Radiation

Studies have also revealed that the substantial correlation between latitude and COVID-19 deaths may be explained by the involvement of sunshine in the production of vitamin D5 by the skin and the studies associating vitamin D insufficiency and COVID-19 fatalities. This demonstrated that the known increase in UV radiation intensity closer to the equator increases the likelihood that populations closer to the equator have more adequate endogenous vitamin D than populations farther away, thereby decreasing the likelihood of fatal immune dysfunction in the presence of COVID-19 [41].

A study by [36] shows that UV rays have direct lethal effects on microorganisms, and as such, the COVID-19 pandemic is seen to be more severe in places with lower UV indices and had a limited lifetime in the open-air dependent on sunlight and humidity. A global analysis study illustrated a smaller effect on seasonal changes in UV rays and their influence on regional patterns of COVID-19 growth rates from January to June [46]. However, there is a need for a comprehensive analysis to understand how COVID-19 cases are influenced by UV rays in all seasons [46]. 

### 3.6. COVID-19 and Air Quality

In Africa, it had been estimated that air pollution would be responsible for 1 to 1 million deaths by 2019. Over 697,000 people died because of air pollution in their homes while 394,000 died because of air pollution in the environment. Deaths caused by air pollution in the environment have been going up, and new records have been set in the continent of Africa [47]. However, most of the deaths linked to ambient air pollution are caused by non-infectious diseases, such as high blood pressure, diabetes, and lung cancer [47]. Air pollution is believed to be a further factor contributing to the high mortality rate associated with COVID-19 transmission. A study in Mozambique determined that the air quality is moderately hazardous, especially in Maputo, where the annual mean concentration of 2.5 micrometer (PM2.5 ) is 21 g/m^3^, exceeding the WHO-recommended maximum of 10 g/m^3^ [39]. In general, the reviewed research indicates that nations with more air pollution (PM2.5) and larger obese populations have a higher probability of severe COVID-19 infection and death [48].

Environmental health problems are getting worse because both outdoor and indoor air pollution is getting worse [49]. However, it has been seen that the level of air pollution inside households is a bigger cause of early death and illness in most parts of Africa. The challenge of inadequate clean energy for households, such as electricity and solar systems, causes high exposure to air pollution, a mixture of fine particulate matter and gases that result from burning fuels inside homes in rural and urban areas with limited ventilation. The environmental health problems existing at the household level due to the burning of solid fuels are the same as being exposed to about PM2.5 pollution outside [15].

As of 2019, the proportion of households using solid fuels for cooking was high in Eastern Africa (95%, UI: 94–95%), Western Africa (83%, UI: 81–86%), and Central Africa (77%, UI: 74–79%) respectively. The lowest proportions were recorded in Southern Africa (32%, UI: 29–35%) [15]

Most people across Africa live in areas with PM2.5 levels that exceed the World Health Organization (WHO) Air Quality Guidelines of 5 g/m^3^, often by a large margin. Particulate matter pollution, such as ambient PM2.5 and air pollution from homes, is suspected to be the leading cause of death in sub-Saharan African countries [49]. Moreover, it is known that about 7 million people die every year from air pollution, making it the fourth leading cause of death in the world [50]. High levels of ground-level ozone accelerated the global spread of COVID-19 [51]. Other studies have established that PM10 was positively linked with daily COVID-19 instances over the study period (PM10: 0.02, 95% CI: (0.06, 0.26); *p* = 0.002) even after the underlying models adjusted for the previously indicated collection of covariables [52].

PM2.5 demonstrated the same trend as the temperature in other parts of Africa with a significant relationship between particulate matter and COVID-19 cases [26,42]. Higher PM2.5 concentrations were related to rapid growth rates when only early outbreaks were considered [26].

Some studies [33] show that four bioclimatic factors (BIO) primarily influence the transmission of COVID-19: isothermality, i.e., day and night temperature (BIO3), precipitation in the wettest month (BIO14), the maximum temperature in the warmest month (BIO5), and mean temperature in the driest month (BIO10), with respective AUCs of 0.618, 0.615, 0.610, and 0.610.

### 3.7. COVID-19 and Land Cover

Land cover is also related to COVID-19 in a variety of ways, including elevation, slope, and aspect, all of which influence the habitability of an area. Therefore, population and socioeconomic activity (such as migration) are determined according to the study by [29]. In addition, the data indicate that an increase of 1 percentage point in the urbanization rate is associated with a 3.1% to 3.7% rise in the number of active COVID-19 cases [52]. The first lesson is recognizing urban planning as a public health activity that requires urgent attention in African cities. The COVID-19 crisis has highlighted the limitations of urban planning for most of Africa’s urban poor in informal settlements and sectors where there is poor sanitation and other essential public health incentives which are important for COVID-19 control [53]. 

## 4. Discussion

This study investigated the impact of environmental conditions on COVID-19 transmission in Africa. The impact of environmental determinants on the transmission of COVID-19 in Africa was demonstrated by a systematic literature review in this study. Studies have been conducted in countries outside of Africa and on a global scale to give policymakers evidence-based research to assist them in responding effectively to the current pandemic [52]. In Africa, the influence of environmental conditions on the transmission of COVID-19 has been the subject of few investigations.

According to the findings of the present study, the available data suggest that COVID-19 shares commonalities with other coronaviruses and the incidence of attacks decrease between the fall and winter. According to Phiri [29], the infection peaked between the months of April, May, June, and July before beginning to decline in August. This is comparable to what is seen in Figure 1. During the months of April, May, June, and July, the number of cases grew in three waves. August 2020 and August 2021 marked the beginning of the case’s decline. The number of fatalities increased during April, May, June, and July in 2020 and 2021. The indication of these results requires that government prepares the safety policies during the cold season, in order to avoid massive infection of the COVID-19 virus [40]. 

It has been demonstrated that the rise in temperature is one of the reasons leading to the low number of COVID-19 transmissions across the majority of Africa [28,31,33,36,43,44]. Similar to other research conducted in Cyprus, it was determined that the rate of exponential growth of COVID-19-infected cases decreases with increasing temperature, reaching zero when the temperature reaches 30.1 ± 2.4 °C [53]. In addition, Chan et al.’s [19] research on previous SARS coronavirus epidemics demonstrated that the virus’ viability was lost at high temperatures (e.g., 38 °C) and high specific humidity (>95%). This result explains why the frequency of cases in various African and Asian nations has decreased in recent months. However, even though this was the result, more research is still needed to figure out why the COVID-19 pandemic reacts differently to the weather conditions in different parts of Africa [32].

Other research findings released by the European Respiratory Society discussed the correlation between temperature and the dissemination of COVID-19 [54]. However, other studies have shown that temperature accounts for only approximately 18% of the variability in illness distribution during the pandemic, and other factors cannot be ignored [37]. The abundance of sunlight in Africa correlates with an increase in the availability of vitamin D, which is vital for protecting against COVID-19 infection by bolstering the immune system. The reported increase in UV light intensity closer to the equator increases the probability that populations closer to the equator have more appropriate endogenous vitamin D than populations farther away, hence decreasing the possibility of a deadly immunological malfunction in the presence of COVID-19 [38]. The majority of nations at risk for vitamin D deficiency are located in South Asia and the Middle East. The efficacy of vitamin D generation in the human body, particularly on the skin, is determined by the degree of sunlight to which the skin is exposed [55]. Africa’s geographic location delivers sufficient UV light for the human body’s health [46].

Air pollution has been identified as a further mechanism contributing to the spread of COVID-19 in highly industrialized nations. Long-term exposure to poor air quality in industrialized nations has increased the incidence of COVID-19 infection [56].

Air pollution is believed to be a further factor contributing to the high mortality rate associated with COVID-19 transmission. Research in Mozambique determined that the air quality is moderately hazardous, especially in Maputo, where the annual mean concentration of PM2.5 is 21 g/m^3^, exceeding the WHO-recommended maximum of 10 g/m^3^ [57]. The World Bank’s estimate for PM2.5 pollution in the air in 2013 was found to be 40% lower than the records for 2019 [15]. This rise shows that the agency needs to put the sources of air pollution in Africa and the rest of the world at the top of its list in order to reduce the risks of COVID-19 and other related diseases spreading. 

It has been reported that nations with greater levels of air pollution (PM2.5) and higher rates of obesity are more susceptible to the transmission of COVID-19 [46]. Moreover, the number of deaths from household air pollution in Africa is higher than the global average of 30 deaths per 100,000 people, which means that Africa has 30% of the world’s disease burden from household air pollution [15]. People in Africa are at a high risk of getting sick and dying from long-term diseases like ischemic heart disease, lung cancer, chronic obstructive pulmonary disease (COPD), stroke, and type 2 diabetes. COVID-19 infection may make these diseases worse [15].

Since the first occurrence, the transmission and severity of the novel coronavirus pandemic have remained relatively low in sub-Saharan nations compared to other regions, such as Europe and the United States [4]. The countries of Sub-Saharan Africa share similar biological, meteorological, and cultural diversity [58]. In 2019, the average number of live births per woman will remain significantly higher than in Europe and North America [59]. The increase in human population mobility caused by international trade has a significant influence on the transmission of highly contagious infectious illnesses, such as COVID-19. Early instances of the pandemic demonstrated geographical diversity across sub-Saharan Africa and locations with a notably high prevalence [60,61]. With the rise of per-urban areas and packed trading areas in Africa, geographical location and land cover will play crucial roles in pandemic and epidemic development [62,63]. Therefore, it is important to ensure that urban planning plays an integral part in public health activity with the capacity to address health crises arising from infectious diseases, such as COVID-19, to improve sanitation and population density [50].

## 5. Conclusions

The information acquired about the impact of climatic and environmental factors on the transmission of COVID-19 in Africa indicates that climatic and environmental factors have an impact on the transmission of COVID-19. The annual epidemic curve illustrates that the number of illnesses increases exponentially with a reduction in temperature during the months of May, June, July, December, January, and February. During these months, the first, second, and third waves had the highest infection and mortality rates. In addition, climatic and environmental parameters, such as temperature, UV radiation, wind speed, humidity air quality, and land cover, have a substantial impact on the transmission of COVID-19. There is a need to ensure that public health interventions are planned to adapt effectively to the climatic and environmental variables that necessitate COVID-19 transmission. Additionally, it is crucial to ensure that proper preparations are made during seasons when the infection incidence is anticipated to be high. Due to the varying seasons encountered in the six regions of Africa, additional research is required to comprehend the climatic and environmental elements at the national level. On a national or regional level in Africa, the cost of the health problems caused by PM2.5 air pollution should be figured out. This includes how much it costs for people to die early or get sick because of air pollution. This will aid policymakers and implementors in the design of globally sustainable interventions. There is a need to enhance remote sensing in epidemiological studies to fully understand the distribution and transmission dynamics and how they correlate with climatic and environmental elements to increase the burden of disease.

## Data Availability

All the data used are available under a creative commons license.

## Appendix B

**Table:**

Author and Year of Study	Study Aim	Study Design	Country	Study Findings	Needs Domain
Sumbana et al., 2020 [60].	To examine the development and potential contributions of risk variables for COVID-19 severity based on Mozambique’s data.	No method used, descriptive Statistics Used	Mozambique	The severity of COVID-19 increases significantly in the elderly and in people with underlying medical disorders, such as cancer, diabetes, obesity, and cardiovascular illness. Another contributing element to the high level of lethality is air pollution.	1. However, there is little COVID-19 data available worldwide to analyze this association and still less in Mozambique. 2. Therefore, the need exists to increase laboratory diagnosis capacity and to monitor compliance of adhering to the intervention measures.
Fernandez et al., 2020 [52].	Our study’s objective is to evaluate the effects of those variables on COVID-19 mortality and geographic dissemination.	A retrospective, observational, longitudinal study	Global	According to the European Public Health Alliance, air pollution is also known to suppress the immune system and impair a person’s capacity to fight against infection. 3.8 million fatalities have been attributed to household air pollution. Our research revealed a connection between COVID-19 and air pollution. However, not all nations experience the same effects because air pollution had a greater influence on HICs and UMICs.	The current COVID-19 pandemic can be associated with the global crises of biodiversity loss and environmental health. Thus, preservation and sustainable management of biodiversity might be necessary to mitigate climate disruption and prevent pandemics to protect health and well-being for the generations to come. Governments plan environmental and health policies as an alternative strategy to respond to new COVID-19 outbreaks and prevent future crises.
Yuan et al., 2020 [30].	The importance of the climate and the environment function considered in transmission	Retrospective	Global	The mean temperature, wind speed, and relative humidity were adversely connected with daily new COVID-19 cases, although the diurnal temperature range was positively correlated. These relationships were more pronounced when the temperature and relative humidity were below normal (21.07 °C and 66.83%, respectively). The wind speed and diurnal temperature range were above average (3.07 m/s and 9.53 °C, respectively). The greatest RR of mean temperature was 1.30 below 23 °C at lag 10 days, the minimum RR of wind speed was 0.29 below 12 m/s at lag 24 days, the maximum RR of temperature range was 2.21 below 28 °C at lag 24 days, and the maximum RR of relative humidity was 1.35 below 4% at lag 0 days. Typically, meteorological influences have a delayed effect on human health. There was a nonlinear correlation between temperature and relative humidity and the number of daily cases. The correlation between humidity and the number of daily instances was negative. When the temperature is greater than 21 °C and the relative humidity is greater than 64%, a modest positive association is observed.	Active measures must be taken to control the source of infection, block transmission, and prevent further spread in all countries. There is relatively little information on the correlation between COVID-19 incidence and wind speed/diurnal temperature ranges globally. The results show that the world pandemic scenario in northern hemisphere countries may increase with the decrease in temperature in the next few seasons, so we should keep vigilant and take active measures. Although, COVID-19 is likely to be less prevalent in the Southern Hemisphere during the summer months, as COVID-19 transmission is influenced not only by meteorological factors, but the epidemic also still requires vigilance. We should take advantage of summer opportunities to prepare for better control of the disease in winter.
Francesco and Rubolini [26].	While adjusting for a number of significant socioeconomic variables and airport links, we examined the effects of climatic conditions (temperature, humidity, and air pollution) on the dynamics of the early COVID-19 outbreak on a worldwide scale between January and May 2020. We demonstrated that COVID-19 growth rates were nonlinearly correlated with climate during the first stage of the global epidemic (January to March) with the quickest spread occurring in locations with a mean temperature of approximately 5 °C and in the most polluted regions.	Statistical analysis, Linear mixed models (LMMs)	Global	The environmental consequences of COVID-19, which spread during the 2020 global pandemic, were not long-lasting and vanished when aggressive containment measures were put in place. When outbreaks that began after mid-March were considered, the effects on the air quality were insignificant, but the effects on the climate lingered for a little longer (until mid-April) but finally vanished as well. Regions with high levels of pollution saw a quicker spread of disease. When later outbreaks were taken into account, the impacts of temperature and air pollution vanished entirely. First, high temperatures, somewhat high humidity, and sunlight reduce the persistence of COVID-19 and other coronaviruses outside the hosts. One of the largest societal challenges is without a doubt controlling COVID-19 outbreaks. Regardless of the climate, policy measures can effectively stop the spread of illness.	To improve the ability of epidemiological models to forecast the risk and time course of future outbreaks and to suggest adequate preventive or containment actions. Total environment ecological regression analyses have identified multiple complex relationships between COVID-19 spread and transmission patterns and diverse environmental features, providing a crucial stimulus to a rapidly evolving area of research. The correlative nature of these analyses should call for cautionary interpretations, as identifying the causal processes linking COVID-19 spread dynamics to environmental features remains challenging.
Salyer et al., 2021 [23].	To assess reported COVID-19 epidemiology data in order to comprehend the spread of the pandemic in Africa.	A cross-sectional study	Africa	For wave analysis, the first wave’s peak weekly incidence (97) with a mean of 18,273 new cases being reported per day for epidemiological week twenty-nine occurred in mid-July 2020. The second wave has not yet reached its apex on the African continent as of December 2020, but the weekly incidence reported for epidemiological week 53 was 129 and the mean number of new cases each day was 23,790. Of the 55 countries, 14 (25%) had only seen or were still seeing their first wave of cases, 40 (73%) had seen or were still seeing their second wave of cases, and four (7%) had seen or were still seeing their third wave of cases as of 31 December 2020.	Promoting efforts that help to better understand the true burden of COVID-19 and the outcomes associated with new circulating COVID-19 variants and their effect on vaccine efficacies are needed, along with campaigns to maintain population practices to prevent the spread of diseases.
Chabane and Arif [27].	The fluctuations of the aerosol optical depth (AOD), black carbon, sulphate, and organic matter in the atmosphere in Blida City, Algeria, which was severely impacted by the COVID-19 pandemic, should be studied, estimated, and discussed.	Following the COVID-19 outbreak in Blida City, which was the most afflicted city in Algeria, we examined the effects of changes in the total AOD, black carbon, sulphate, and organic matter in the atmosphere (=550 nm) in the same period of 2019 and 2020.	Algeria	The quarantine that was implemented to stop the spread of COVID-19 had negative impacts that could be seen in the overall AOD as well as certain atmospheric components. When these factors were compared between 2019 and 2020 (during the quarantine months), it became clear that in April, the BCAOD values in 2020 were significantly lower than those in 2019. Low-level sulphur dioxide inhalation can make chronic respiratory conditions, such as asthma and emphysema, worse. Inhaling sulphur dioxide or consuming foods preserved with sulphates might trigger lung spasms in some asthmatics.	The variable related to aerosol optical depth (AOD) is sensitive to multiple pollutants in the atmosphere, including black carbon, sulphur, and organic materials. It is therefore an appropriate variable to study the transmission of viruses owing to pollution levels around the communities or geographical areas.
Sharif et al., 2020 [36].	To assess the relationship between environmental variables and the COVID-19 pandemic and its epidemiology across nine nations and five continents.	Descriptive statistical analysis was conducted for the study regions.	Global	Environmental factors and COVID-19 survival were found to be statistically associated. Between 12 March 2020 and 30 June 2020, there were 61,172 cases and 1082 fatalities in South Africa’s Western Cape, which is where the majority of cases in Africa were discovered. Daily average temperatures were 15 °C, relative humidity was 70%, UV index was 7 on average, and wind speed was 17 km/h. UV light destroyed microbes directly. The severity of the COVID-19 pandemic exacerbated in regions with lower UV indices. The average UV index for each day was calculated and displayed together with the number of cases and fatalities. In the Western Cape, 87% of cases and 93% of fatalities were found at 5.5 mean UV per day.	This study claims that, if necessary steps are not taken, the COVID-19 pandemic will become worse in the world during the winter days ahead with reduced temperature.
Yuan et al., 2020 [38].	The effects of meteorological factors on daily new cases of COVID-19 in 127 countries as of 31 August 2020.	The log-linear generalized additive model (GAM) was used to analyze the effect of meteorological variables on daily new cases of COVID-19.	Global	In over 127 nations where temperature, relative humidity, and wind speed were below 20 °C, 70%, and 7 m/s, respectively, there may be a negative correlation between temperature, relative humidity, and wind speed and daily new COVID-19 cases. Daily new instances were strongly connected with temperature above 20 °C and relative humidity above 70%; however, the latter correlation was not strong. There was no statistically significant correlation between COVID-19 transmission and wind speed (above 7 m/s).	The basis of concluding about the effect of meteorological conditions on the transmission of COVID-19 is still controversial. To date, several studies examining the effects of meteorological variables on COVID-19 transmission have explored the role of temperature and other related climatic variables.
Yaro et al., 2020 [33].	The effect of demographic and environmental variables on the transmission of severe acute respiratory syndrome coronavirus 2 (COVID-19) in Nigeria.		Nigeria	The bioclimatic variables that had a significant impact on COVID-19 transmissibility in Nigeria included oscillation in day and night temperature (isothermally—BIO3), precipitation of the wettest month (BIO14), maximum temperature of the warmest month (BIO5), and mean temperature of the driest month (BIO10). The transmission rate of COVID-19 increases with increasing precipitation whereas it decreases with increasing temperature.	Therefore, there is a need for relevant stakeholders in the health sector to adopt a more collective and integrative approach to the control of the virus.
Adekunle et al., 2020 [34].	To determine whether findings on the climatic circumstances of COVID-19 growth are regionally specific by looking at the impact of meteorological indicators on the development or not of coronavirus infections in Africa.	We rely on the generalized additive model (GAM).	Africa	The growth curve of COVID-19 in Africa is inversely correlated with both the mean temperature and the average wind speed. According to our research, there is no statistically significant correlation between relative humidity and the COVID-19 exposure-response curve in Africa. The crucial functions of wind speed and mean temperature in promoting and inhibiting COVID-19 growth in Africa, respectively, are explained by this study. In underdeveloped African nations with little access to clean water sources, social isolation and handwashing may be challenging; however, weather conditions that make COVID-19 less likely to survive may make up for these drawbacks.	The study is limited to the obtained findings permitted by the GAM model. Another pervasive limitation of this study could be traced to regional differences in testing rates, political interests to withhold information on COVID-19 cases and deaths, unavailability of data on non meteorological covariates, limited health system services and different travel patterns and contact rates with people from other continents.
Matthew et al., 2021 [35].				The spatial distribution of the illnesses revealed that the disease spread more quickly in the northern hemisphere (high latitudes, temperate or continental climate) with Europe and North America being the main destinations for the transmission. The findings showed that different climatic regions of the world saw varying effects of daily climatic fluctuations on the transmission of COVID-19 infection. In approximately 42 (68.85%) of the chosen nations, we discovered strong negative (0.510 r 0.967) and positive (0.519 r 0.9999) associations between climatic factors and confirmed COVID-19 instances. In 49.2% of the nations that were chosen, there were significant correlations between temperature and other variables (with 34.4% positive correlations and 14.6% negative correlations).	It should be seriously noted that climatic factors are not the only variables responsible for the observed variations in disease transmission. Further areas of research can look at the effects of climate on the spread of the disease while controlling for other factors that can affect transmissibility, such as adherence to COVID19 control measures, international travel, and population density
Notari et al., 2020 [40].	In the month of the beginning epidemic growth, we look for a correlation between the rate and the average temperature T of each country.		Global	Finally, additional environmental variables, including humidity, wind speed, air pressure, and pollution, may also be important. Since other coronaviruses have a similar decline at high temperatures, the drop is expected. The drop at low temperatures (less than 8 °C), which is included in the base dataset, is questionable though.	The limitation of the study is the use of average temperature, which is not very accurate for large countries, especially those that have a large spread in latitude and climatic conditions.
Lulbadda et al., 2020 [37].	The main goal of this research is to better understand how COVID-19 dissemination relates to environmental factors including temperature, population density, median age, and healthcare facilities.	The relationships between the variables and COVID-19 cases during the study periods were determined using a negative binomial regression model.	Experimental	According to the findings, when considered separately in the model, population size, average temperature, and median age do not have a significant influence. They do, however, have a considerable impact on the number of instances when combined with other variables.	With the reported correlation between climatic and COVID-19 cases, it is difficult to explain precisely how the number of cases changes for a unit increase in each of the variables because that change depends on different values taken by other variables as well.
Mashrur F.R et al., 2021 [49].	To enable policymakers to take proactive actions for the upcoming waves, it is important to identify the most important risk factors for spreading COVID-19.	Cross-section study	Global	Air pollution, PM2.5, the number of days to impose lockdown from the first case (r = 0.38, *p* = 0.0424), the total confirmed cases on the first lockdown (r = 0.61, *p* = 0.0004), and the number of days to impose lockdown from the first case were associated with outcome measures in the correlation analysis. The most important exposure variables for the spread of COVID-19 in the adjusted model were air pollution (l = 4.5, *p* = 0.0127, |t| = 3.1) and overweight prevalence (l = 4.7, *p* = 0.0187, |t| = 2.9).	The reported cases might not be the entire picture or representation of the COVID-19 situation in a country. Many affected patients remain undetected, making it too hard for them to get a sense of accurate total case information of a country. Second, the sample size used in the study was small. Future research should increase the number of countries. Third, we had only considered countries that crossed the peak of active cases from the curve until 10 June 2020.
Nguimkeu P and Tadadjeu S, 2020 [53].	Intends to examine the influence of geographic and demographic (DG) characteristics in the SSA’s lower level of epidemic severity than other regions.	Cross-section Study	Sub-Saharan African Countries	We discovered that while the average temperature around the first quarter of the year (January–March) is negatively associated with this epidemic outcome, the percentage of the population 65 years and older, population density, and urban population rate are all positively associated with the number of active cases. Sub-Saharan African nations are less impacted by these causes than other nations since they have higher levels of the latter and lower rates of the former. Therefore, compared to the rest of the globe, these characteristics are found to have smaller marginal effects on the number of active cases in sub-Saharan Africa.	The limitation of the analysis is the quality of the publicly available data that was used and the associated misreporting or underreporting in the outcome variable. Econometric approaches to deal with these issues such as the one employed may not fully mitigate it or fully identify some relevant components of the relationship, especially if the measurement errors are correlated with explanatory factors. It is worth noting another important limitation, which is the inability of the model to measure the endogenous behavioral responses of some of the key explanatory variables.
Phiri D et al., 2021 [29].	Understanding the relationship between COVID-19 instances in Zambia, a sub-Saharan African nation, and environmental and socioeconomic aspects	The dataset was organized, extracted, and established using geospatial methods, and the factors connected to COVID-19 instances were examined using a classification tree (CT) technique.	Zambia	The findings demonstrated that socioeconomic variables as opposed to environmental ones significantly influenced the distribution of COVID-19 cases in Zambia. More specifically, the binary model revealed that the proximity to the airport, the density of the population, and the distance to the town centers were the factors that combined to have the greatest influence while the risk level analysis revealed that regions with higher rates of the human immunodeficiency virus (HIV) infection had a disproportionately higher likelihood of having a high number of COVID-19 cases than regions with lower HIV rates. Districts with lower COVID-19 case probabilities are those that are far from large urban centers and have hotter weather.	The data used was accessed at the district level, and hence some of the details might not have been captured because they need small mapping units. Furthermore, it was not possible for the data to include human behavior attributes, age group, and socioeconomic situation because of limited access to detailed information on COVID-19 patients. Second, the number of COVID-19 cases has continued to rise, especially during the cold season (July and August), and this is likely to affect the patterns and the distribution of COVID-19 cases. As such, these results might not be replicated or might vary if datasets for later dates are used, yet they remain relevant to use in controlling the surge of COVID-19 cases in similar situations. Third, the factors considered in this study do not represent all the potential factors that can be considered. Finally, due to the limitation in testing facilities and the logistics in fighting the COVID-19 pandemic, all the districts did not have the same testing facilities and opportunities.
Chen et al., 2021 [39].	Multivariable regression analyses.	Visual examination of globe maps reveals that coronavirus disease 2019 (COVID-19), where heat and humidity are prone to be higher, is less common in nations closer to the equator.	Global	According to our findings, a nation that is 1000 km closer to the equator may anticipate 33% fewer cases per million people. Since the Earth’s angular tilt changes by approximately 23.5° between the equinox and the solstice, one may anticipate a difference of 64% in cases per million people between two hypothetical nations whose climates vary by about as much as two nearby seasons. Our findings indicate that new COVID19 cases are predicted to decrease throughout the summer and increase during the winter in various nations. Our revised datasets contain larger numbers of observations. A 4.3% rise in COVID19 cases per million people is correlated with a 1° increase in absolute latitude. Summertime temperatures rising and prolonged exposure to sunlight may increase	It is worth noting that our findings are consistent with the generated hypothesis that higher temperatures and more intense UV radiation reduce COVID-19 transmission; the precise mechanisms for such an effect remain unclear and may indeed comprise not only biological but also behavioral factors which need to be accounted for. Thus, future research should aim at uncovering how the transmission of COVID-19 is affected by changes in (1) climatic factors, such as heat and humidity, (2) geographic factors, such as altitude and sunlight intensity, (3) factors related to human behavior, such as social interactions and pollution due to local economic activity at a more disaggregated level, and (4) the different potential of the human immune system to cope with diseases in summer as opposed to winter. Second, even though we included all countries worldwide for which data for this analysis were available, our final dataset included only 117 out of the world’s countries, for reasons of data availability and for some countries not yet having surpassed the 100 COVID-19 case threshold.
Diouf et al., 2022 [32].	Examining the possible impact of climate factors on COVID-19 transmission over sixteen carefully chosen nations in three different climate zones in Africa (the Sahel, the Maghreb, and the Gulf of Guinea).	Correlation	Africa	The findings show inverse associations between COVID-19 instances and temperature over the Maghreb and Gulf of Guinea regions that are statistically significant. Positive relationships, on the other hand, are discovered throughout the Sahel region, particularly in the central region, which includes Niger and Mali. The Sahelian and Gulf of Guinea countries show significant and positive values in correlations with certain humidity and water vapor parameters while the countries of the Maghreb show significant and negative values. The three climatic zones’ respective influences on the COVID-19 pandemic transmission are (i) the cold and dry conditions over the Maghreb; (ii) the warm and humid conditions over the Sahel; and (iii) the cold and humid conditions over the Gulf of Guinea.	The findings of the study indicate the need for further studies to investigate why the COVID-19 pandemic has different sensitivities to the climate conditions observed across the three climatic regions.
Cambaza et al., 2020 [31].	To analyze the relationships between weather and the frequency of confirmed COVID-19 cases in Mozambique, Southern Africa	Correlation	Africa	All areas showed negative correlations between temperature and the number of cases while Nampula Province and the Maputo region showed positive correlations. A bubble chart made it possible to visualize the relationship between the two weather variables and the overall number of cases, which suggests that the number of cases rises as pressure and temperature fall. In Mozambique, the number of confirmed cases of COVID-19 seems to be linked to the temperature and air pressure.	Decision makers should consider the weather as a predictor of the rate at which the pandemic is spreading in the country.
Aidoo et al., 2021 [43].	The effect of some local weather variables (average temperature, average relative humidity, average wind speed and average atmospheric pressure) on the risk of Severe Respiratory Syndrome Coronavirus 2 (SARS-CoV-2) in Ghana	GAM, semiparametric extension of generalized linear model (GLM)	Africa	Wind speed and pressure have a positive linear relationship with the spread risk of COVID-19 while temperature and humidity have a nonlinear relationship with the spread of COVID-19.	The need for policymakers to design effective countermeasures for controlling the spread as we are still within the low temperature season.
Mbandi M. A, 2020 [50].	Air pollution in Africa in the time of COVID-19: the air we breathe indoors and outdoors	Commentary	Africa	With the reduction in air pollution due to lockdown measures in place during the COVID-19 pandemic, it seemed like indoor air quality was the major risk to African homes.	Despite the COVID-19 pandemic, there is still a lot of room for improvement in Africa when it comes to surveillance systems and hygiene. Moreover, as there is more evidence that COVID-19 is spreading in Africa, modeling must take into account environmental factors, such as air quality, trends in fuel use, and the link between disease outbreaks.
Reche et al., 2020 [45].	To demonstrate that even in pristine environments, above the atmospheric boundary layer, the downward flux of viruses ranged from 0.26 × 10^9^ to >7 × 10^9^ m^−2^ per day.	Experimental, MTX ARS 1010 automatic deposition collectors	Global	The daily deposition rates of viruses associated with aerosols < 0.7 μm in size explain observations that identical viral sequences occur at geographically distant locations and in very different environments. There is evidence that bacteria and viruses can still live after being carried by the air, which fits with the fact that microbes can be found in very different ecosystems.	The large amounts of bacteria and viruses that fall from the atmosphere may change the structure and function of ecosystems that they reach. These effects should be further investigated.
Meo et al., 2020 [28].	To investigate the impact of weather conditions, heat, and humidity on the incidence and mortality of the COVID-19 pandemic in various regions of Africa	One-way ANOVA and correlation coefficient	Africa	In African countries, an increase in relative humidity and temperature was associated with a decrease in the number of daily cases and deaths due to the COVID-19 pandemic. Poisson regression results showed that with a 1% increase in humidity, the number of cases and deaths decreased (=−0.037, S.E. = 0.0001, *p* < 0.001) and deaths (β = −0.035, S.E. = 0.0004, *p* = 0.001) were significantly reduced by 3.6% and 3.7%, respectively.	Regional epidemiological trends and weather events related to the COVID-19 pandemic should be easier to predict. This will help the public be more aware and ready to take more appropriate steps and will help plan for the future to fight against pandemics.
Ogunjo et al., 2022 [42].	Investigated the role of temperature, relative humidity, and particulate matter in the spread of COVID-19 cases within two densely populated cities of South Africa—Pretoria and Cape Town.	Linear and quantile regression and Granger Causality Test	South Africa	This study has shown that the effect of meteorological variables, especially temperature and relative humidity, is not the same everywhere. This suggests that other variables could be causing the causal relationships in places like Cape Town where they were seen. There was a significant relationship between particulate matter and COVID-19 in the two cities. Based on the significance of the causality test, particulate matter was found to be a good predictor of COVID-19 cases in Pretoria with a lag of seven days or more.	There is still a need for further studies, most importantly, when we have longer time series to unravel the role of meteorological data in COVID-19 transmission. Temperature and relative humidity are factors that should be given special attention, especially in the hinterlands.
Fisher et al., 2020 [48].	To quantify how air pollution is affecting health, human capital, and the economy across Africa with a particular focus on Ethiopia, Ghana, and Rwanda.	Estimated economic output lost due to air pollution-related disease by country with use of labor income per worker, adjusted by the probability that a person (of a given age) was working	Africa	Air pollution was responsible for 1–1 million deaths across Africa in 2019. Household air pollution accounted for 697,000 deaths and ambient air pollution for 394,000. Ambient air pollution-related deaths increased from 361,000 in 2015 to 3,831,000 in 2019 with the greatest increases in the most highly developed countries. The majority of deaths due to ambient air pollution are caused by non-communicable diseases. The loss in economic output in 2019 due to air pollution-related morbidity and mortality was $3.02 billion in Ethiopia (1.16% of GDP), $1.63 billion in Ghana (0.95% of GDP), and $349 million in Rwanda (1.19% of GDP). PM25 pollution was estimated to be responsible for 196 billion lost IQ points in African children in 2019.	Courageous and visionary leaders who recognize the growing danger of ambient air pollution, engage civil society and the public, and take bold, evidence-based action to stop pollution at the source will be key to the prevention of air pollution in Africa.
Health Effect Institute, 2022 [15].	Reports an overview of the state of air quality and its impact on health in Africa.	Compilation of disease project and from a recent global assessment of air pollution sources to discuss air pollution trends, sources, and associated disease burdens across this important region, with a particular focus on Egypt, Ghana, Democratic Republic of the Congo, Kenya, and South Africa.	Africa	In 2019, air pollution contributed to 1.1 million deaths in Africa. Of these, more than 63% were linked to exposure to household air pollution. In Africa, air pollution is the second-leading risk factor for deaths. Countries in Africa experience some of the highest PM2.5 exposures in the world. Although a lack of monitoring stations makes estimates uncertain, limited monitoring and modeled estimates indicate that most people in Africa breathe unhealthy levels of PM2.5 pollution. The burden of disease from household air pollution in Africa is among the highest in the world.	Most studies on the effects of air pollution on health in Africa have looked at respiratory health, but some have also looked at heart health or the health of mothers and children. Need for Comprehensive Air Pollution and Health Studies in Africa.
World Bank, 2022 [51].	This publication aims to further contribute to the evidence base on air-quality management by providing up-to-date estimates of the global economic costs of air pollution. The analysis builds on previous estimates by the Bank and its partners and is based on cutting-edge scientific findings of the health effects of air pollution e comprehensive air-quality data from monitoring stations in a large number of cities across the world.	This report uses the GBD 2019 estimates of premature mortality and morbidity attributable to PM2.5 air pollution to value the economic cost in dollar terms.	Global and Region	There is no doubt that prolonged exposure to particulate matter pollution, particularly PM2.5, is linked to an increase in mortality from all causes, particularly cardiovascular causes, even at exposure levels below the 10 g/m^3^ or PM2.5 annual exposure level that is currently recommended by the WHO (Chen and Hoek, 2020). There is strong evidence showing a robust, positive association between short-term exposure to PM10, PM2.5, NO_2_, and O_3_ and all-cause mortality, and between PM10 and PM2.5 and cardiovascular, respiratory, and cerebrovascular mortality (Orellano et al., 2020). Short-term exposure to sulfur dioxide (SO_2_), ranging from increases in exposure from one hour to a 24 h average, is robustly associated with increased mortality (Orellano, Reynoso, and Quaranta, 2021).	As scientific research continues to evolve, there is a high probability that evidence will show that air pollution’s health and economic burdens are even higher than in this report. Firstly, it indicates the importance of prioritising efforts to reduce air pollution emissions from coal-fired power plants and diesel-fueled vehicles because the particles in those emissions are more damaging to health than particles from most other air pollution sources (Thurston, Awe, Ostro, and Sánchez-Triana, 2021). Secondly, it demonstrated that particulate matter from dust should continue to be factored into global estimates of the burden of disease from air pollution given the substantial health impact of dust (Ostro, Awe, and Sánchez-Triana, 2021). Third, it makes a strong case for increasing efforts to set up ground-level networks to monitor air quality in low- and middle-income countries by showing that satellite-based estimates of air quality are not as accurate as data from the ground (World Bank, 2021).
